# Immunofluorescence reveals neuron-specific promoter activity in non-neuronal cells

**DOI:** 10.17912/8FDA-CK77

**Published:** 2018-07-20

**Authors:** Sierra K. Lear, Alakananda Das, Miriam B. Goodman

**Affiliations:** 1 Chemical & Biomolecular Engineering, Tulane University, New Orleans, LA; 2 Molecular and Cellular Physiology, Stanford University, Stanford, CA

**Figure 1.**
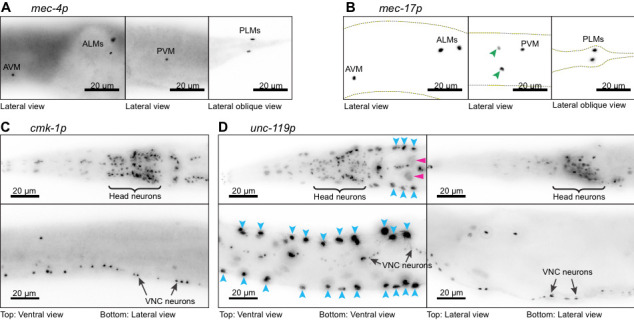


## Description

Four transgenes designed to drive expression of Cre recombinase and a nuclear-localized blue fluorescent protein (*<Neuron-specific promoter>::Cre::F2A::BFP::AID::NLS::tbb-2 3’UTR*) were expressed under the control of neuron-specific promoters. All transgenes were inserted into the *ttTi5605* transposon insertion site in chromosome II of wild-type *C. elegans* animals (N2) using CRISPR/Cas9-mediated genome editing, equalizing any potential positional effects. We observed very faint BFP signal in live worms from the signal copy transgene insertions. Additionally, the signal was further masked by bright autofluorescence from the intestine. To observe the cells expressing these transgenes we performed immunofluorescence experiments using primary antibody against BFP. Using this assay, we studied the expression from touch receptor neuron (TRN)-specific promoters, *mec-4p* (A) and *mec-17p* (B) and pan-neuronal promoters, *cmk-1p* (C) and *unc-119p* (D). As expected, BFP expression driven by the *mec-4*promoter is observed exclusively in the six TRN nuclei (A). However, under *mec-17* promoter, BFP expression is observed in two additional neuronal nuclei (B, green arrowheads) besides the six TRN nuclei (B). These two neurons are likely to be PVD or PDE neurons based upon the location of their nuclei relative to the PVM nucleus. In case of the pan-neuronal promoters, expression under *cmk-1* and *unc-119* promoters is observed in most, if not all neuronal nuclei, including in many head neurons (C and D top) and ventral nerve cord (VNC) neurons (C and D bottom). However, in a subpopulation of animals expressing BFP under the *unc-119* promoter, intense fluorescence was observed in several additional larger nuclei along the lateral sides (D, blue arrowheads), which we identify as hypodermal cells and faint signals in even larger nuclei (D, pink arrowheads), which we identify as intestinal cells. We did not observe any fluorescence in our control animals (N2) which were treated similarly (data not shown). While this experiment confirmed expected expression patterns for *mec-4p* and *cmk-1p*, additional expression in other neurons for *mec-17p* and other tissue types in *unc-119p* promoters could reflect the specific promoter sequences used in this study or that low levels of expression in these unexpected cells were not readily detected from direct observation of fluorescent protein expression analyzed previously.

## Methods

All worm strains were grown at 20 °C on NGM plates containing OP50-1. Well-fed adult hermaphrodites were fixed according to the protocol described by Michael Koelle (1), with the following modifications:

1. All steps after the initial washing off worms from the NGM plates were performed in 48-well plates instead of microcentrifuge tubes.
2. Instead of centrifugation, worms were allowed to settle by gravity to reduce shear-induced rupturing of worms.
3. Primary antibody against BFP (rabbit polyclonal antibody, Evrogen AB233) was used at 1:5000 dilution.
4. F(ab’)2-Goat anti-rabbit IgG (H+L) secondary antibody, Alexa Fluor® 568 conjugate (Invitrogen, A-21069), was used at 1:4000 dilution.

## Reagents

The following strains are used in this study. GN706 *(pgSi101[mec-4p::Cre::F2A::BFP::AID::NLS::tbb-2 3’UTR]) II*GN707 *(pgSi102[mec-17p::Cre::F2A::BFP::AID::NLS::tbb-2 3’UTR]) II*GN751 *(pgSi114[cmk-1p::Cre::F2A::BFP::AID::NLS::tbb-2 3’UTR]) II*GN730 *(pgSi110[Cbr-unc-119p::Cre::F2A::BFP::AID::NLS::tbb-2 3’UTR]) II*

The following promoter sequences was used in this study.*mec-4p*: 1020 bp region immediately upstream of *mec-4* start codon (2)*mec-17p*: 868 bp region immediately upstream of *mec-17* start codon*cmk-1p*: 2000 bp region immediately upstream of *cmk-1* start codon (3)*Cbr-unc-119p*: 988 bp region immediately upstream of *Cbr-unc-119* start codon (4)

Full sequences of all reported alleles are available upon request.

## References

[R1] Koelle, M. 1998. Antibody staining of C. elegans. https://medicine.yale.edu/lab/koelle/protocols/Antibody%20Staining_180540_21947_v1.pdf

[R2] Royal DC, Bianchi L, Royal MA, Lizzio M Jr, Mukherjee G, Nunez YO, Driscoll M (2005). Temperature-sensitive mutant of the Caenorhabditis elegans neurotoxic MEC-4(d) DEG/ENaC channel identifies a site required for trafficking or surface maintenance.. J Biol Chem.

[R3] Schild LC, Zbinden L, Bell HW, Yu YV, Sengupta P, Goodman MB, Glauser DA (2014). The balance between cytoplasmic and nuclear CaM kinase-1 signaling controls the operating range of noxious heat avoidance.. Neuron.

[R4] Frøkjær-Jensen C, Davis MW, Ailion M, Jorgensen EM (2012). Improved Mos1-mediated transgenesis in C. elegans.. Nat Methods.

